# Exploring CJD incidence trends: insights from Slovakia

**DOI:** 10.1080/19336896.2024.2349011

**Published:** 2024-05-09

**Authors:** Pavol Skacik, Egon Kurca, Stefan Sivak

**Affiliations:** a Neurology Department, University Hospital Martin, Martin, Slovakia; b Jessenius Faculty of Medicine in Martin, Comenius University Bratislava

**Keywords:** Creutzfeldt-Jakob disease, geographical cluster, human prion diseases, incidence, Slovakia

## Abstract

Authors are commenting on the evolving geographical incidence trends observed with the genetic form of Creutzfeldt-Jakob disease and discussing the diverse array of factors contributing to the heightened incidence rates observed in specific geographical regions.

Dear Editor,

We have carefully reviewed the research paper titled “A systemic analysis of Creutzfeldt Jakob disease cases in Asia” published by [1] 18:1, 11–27. First and foremost, we extend our congratulations of the success of this noteworthy research paper and would like to contribute additional information.

One topic discussed in this paper is the incidence of the genetic form of Creutzfeldt-Jakob Disease (CJD) in China. The E200K mutation emerges as the second most prevalent mutation, exhibiting a geographical distribution pattern and predominance in the northern regions of China, specifically north of the Yangtze River.

In Slovakia, a captivating focal clustering of genetic CJD has been observed in select regions, with a national incidence rate of 3 cases per 1,000,000 inhabitants [2]. In our recent epidemiological study conducted in the northern sector of central Slovakia, encompassing a population of approximately 223,000 individuals over the period from 2006 to 2023, we observed an incidence rate of 13 cases per 1,000,000 persons. A predominant proportion of the observed cases manifested as genetic CJD with positive E200K mutation. Certain localized regions within this area exhibited an elevated incidence, reaching up to 17 cases per 1,000,000 individuals [3].

The diverse array of factors contributing to the heightened incidence rates observed in specific regions is multifaceted. Primarily, genetic clustering may be attributable to the founder effect within the populace inhabiting these areas. Furthermore, potential influences from environmental factors have been considered [4]. Moreover, advancements in understanding this disease, combined with heightened clinical suspicion and knowledge among healthcare providers for individuals exhibiting symptoms of rapidly progressive dementia syndrome, may be associated with a higher diagnosis rate. Diagnostic modalities, encompassing magnetic resonance imaging, EEG, lumbar puncture, genetic analysis, and real-time quaking-induced conversion (RT-QuIC), have played pivotal roles in facilitating the detection and characterization of Creutzfeldt-Jakob Disease (CJD).

Overall, this research contributes significantly to our understanding of epidemiology of CJD and emphasizes the importance of continued surveillance, research, and collaboration in addressing this neurological condition.

## Authors contribution

All authors contributed to manuscript revision and approved the final version for submission. All authors agree to be accountable for all aspects of the work.

Pavol Skacik: Writing – Review and Editing.

Stefan Sivak: Drafting of the paper.

Egon Kurca: Conception and design.

## Data Availability

Data sharing is not applicable to this article as no new data were created or analysed in this study.
